# Multiple receptor tyrosine kinases are expressed in adult rat retinal ganglion cells as revealed by single-cell degenerate primer polymerase chain reaction

**DOI:** 10.3109/03009731003597119

**Published:** 2010-03-10

**Authors:** Niclas Lindqvist, Ulrika Lönngren, Marta Agudo, Ulla Näpänkangas, Manuel Vidal-Sanz, Finn Hallböök

**Affiliations:** ^1^Department of Neuroscience, Unit for Developmental Neuroscience, Biomedical Center, Uppsala University, 75123 UppsalaSweden; ^2^Departamento de Oftalmología, Facultad de Medicina, Universidad de Murcia, MurciaSpain; ^3^Fundación para la Formación e Investigación Sanitaria de la Región de Murcia, Hospital Universitario Virgen de la Arrixaca, MurciaSpain

**Keywords:** Receptor tyrosine kinase, retinal ganglion cell, single-cell analysis

## Abstract

**Background:**

To achieve a better understanding of the repertoire of receptor tyrosine kinases (RTKs) in adult retinal ganglion cells (RGCs) we performed polymerase chain reaction (PCR), using degenerate primers directed towards conserved sequences in the tyrosine kinase domain, on cDNA from isolated single RGCs univocally identified by retrograde tracing from the superior colliculi.

**Results:**

All the PCR-amplified fragments of the expected sizes were sequenced, and 25% of them contained a tyrosine kinase domain. These were: Axl, Csf-1R, Eph A4, Pdgfrβ, Ptk7, Ret, Ros, Sky, TrkB, TrkC, Vegfr-2, and Vegfr-3. Non-RTK sequences were Jak1 and 2. Retinal expression of Axl, Csf-1R, Pdgfrβ, Ret, Sky, TrkB, TrkC, Vegfr-2, and Vegfr-3, as well as Jak1 and 2, was confirmed by PCR on total retina cDNA. Immunodetection of Csf-1R, Pdgfrα/β, Ret, Sky, TrkB, and Vegfr-2 on retrogradely traced retinas demonstrated that they were expressed by RGCs. Co-localization of Vegfr-2 and Csf-1R, of Vegfr-2 and TrkB, and of Csf-1R and Ret in retrogradely labelled RGCs was shown. The effect of optic nerve transection on the mRNA level of Pdgfrβ, Csf-1R, Vegfr-2, Sky, and Axl, and of the Axl ligands Gas6 and ProteinS, was analysed. These analyses show transection-induced changes in Axl and ProteinS mRNA levels.

**Conclusions:**

The repertoire of RTKs expressed by RGCs is more extensive than previously anticipated. Several of the receptors found in this study, including Pdgfrβ, Csf-1R, Vegfr-2, Sky, and Axl, and their ligands, have not previously been primarily associated with retinal ganglion cells.

## Background

The family of receptor tyrosine kinases (RTKs) consists of more than 50 different transmembrane polypeptides with an intracellular tyrosine kinase domain (1,2). The majority of the characterized RTKs are receptors for protein ligands and mediate a variety of responses in eukaryotic cells such as proliferation, differentiation, motility, and survival. Ligands that bind RTKs usually induce receptor dimerization and autophosphorylation with a subsequent activation of several signal transduction pathways (3). Retinal ganglion cells (RGCs) are located in the outermost layer of the retina and are the sole retinal neurons that send their axons, that form the optic nerve, outside the eye to convey the visual information to the brain visual centres. In rodents, the majority of RGCs project to the superior colliculi, and thus RGCs can be univocally identified in the retina by applying retrogradely transported neuronal tracers, such as FluoroGold or True Blue, to the superior colliculi, as the tracers will be transported through the RGC axons to their soma, which lays in the retina (4,5).

During development, RTKs are involved in the regulation of RGC neurite growth, axon guidance, and naturally occurring cell death. In the postnatal nervous system, the function of RTKs is less clear. Data show that RTKs play roles in regulating neuronal plasticity (4). RTKs have also been shown to mediate support for adult injured RGCs. RGC axotomy, i.e. transection of the optic nerve, leads to the death of RGCs, and approximately 90% of them are lost by day 14 post-axotomy (6,7). The RTK TrkB is expressed by RGCs, and intra-ocular injection of its ligand, brain-derived neurotrophic factor (BDNF), postpones the death of axotomized RGCs alone (8) or together with glial cell-line-derived neurotrophic factor (9). In the postnatal animal the number of many central nervous system neurons including RGCs is normal in knock-out mice for both TrkB and BDNF (10,11). However, neurons are more prone to die in mice with double gene knock-out for RTKs. Double knock-out of TrkB and TrkC, the receptor for neurotrophin-3, leads to increased death of central neuronal populations, which do not die in either of the single gene knock-out mice (12). Single and double gene knock-out of members of the Axl, Sky/Tyro-3, Mer-RTK subfamily have mild phenotypes, while the triple mutant exhibited increased apoptosis and neuronal degeneration (13,14). This suggests that central neurons have the capacity to utilize several factors, and certain neurons may even be dependent on several factors for their normal development and maintenance (15).

**Figure 1.**
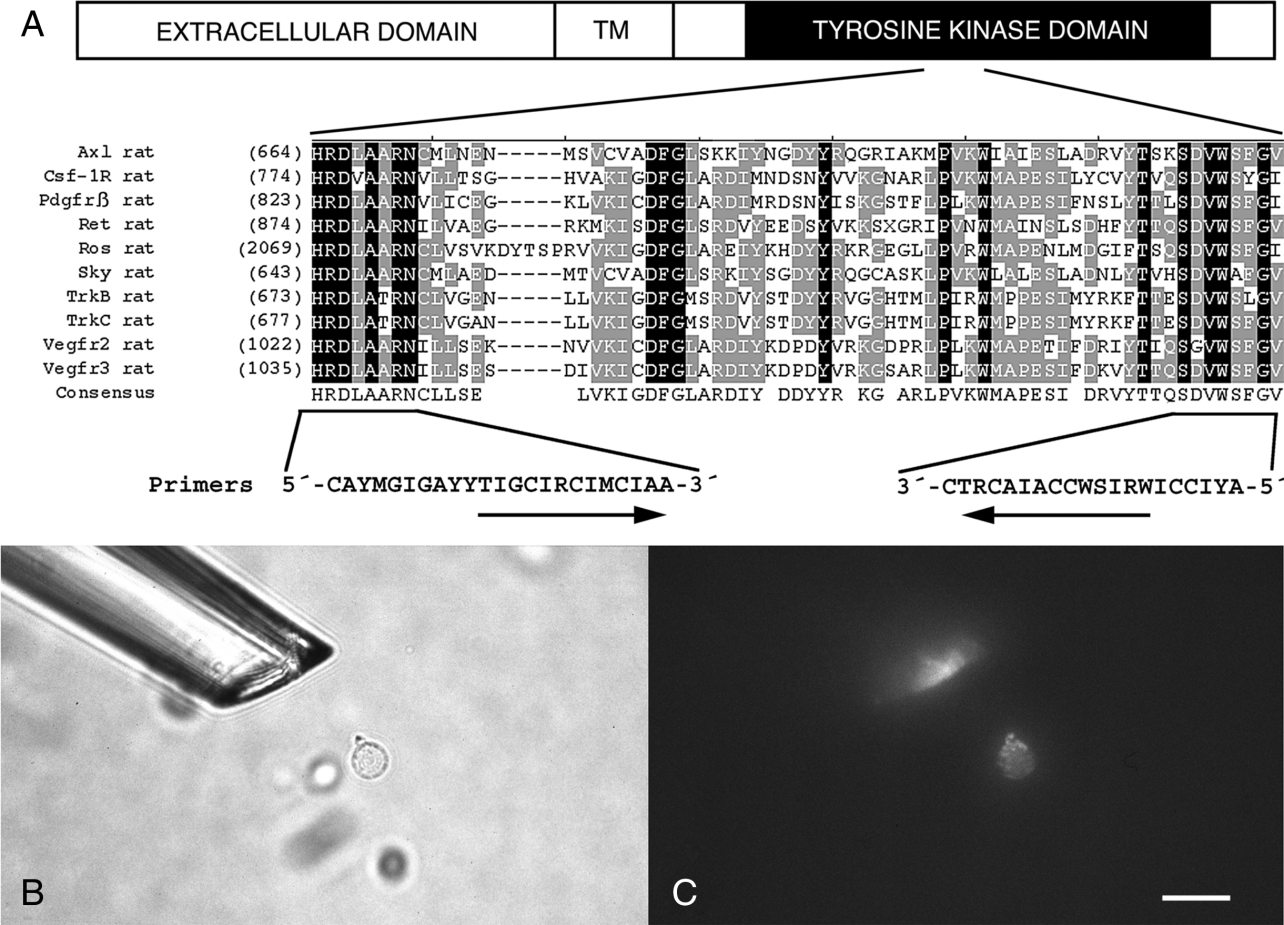
Design of degenerate primers for PCR and single retinal ganglion cell isolation. A: Schematic diagram of receptor tyrosine kinases (RTKs) and degenerate primers with alignment of the amino acid sequences in conserved regions of the tyrosine kinase domain (HRDLAARN and DVWS(F/Y)G(V/I)) in the identified RTKs, which were used for the primer design. The oligonucleotide sequences are shown in international union of biochemistry (IUB) nucleotide codes. Identical conserved amino acids are boxed in black and less well conserved in grey. Bright-field (B) and fluorescence micrographs (C) showing a labelled single retinal ganglion cell in process for aspiration. Scale bar 20 μm valid for B and C.

The aim of this study was to identify RTKs expressed in adult rat RGCs. The retinal ganglion cell layer (GCL) harbours both RGCs and displaced amacrine cells, which complicates the analysis. To exclude displaced amacrine cells and to identify RGCs we used retrograde tracing of RGCs from the superior colliculi with fluorescent tracers. The RGCs were subsequently isolated using a mechanical layer separation technique followed by microaspiration of single fluorescent RGCs (16). We used quantitative reverse transcriptase polymerase chain reaction (Q RT PCR) to amplify RTK sequences from the aspirated single RGCs using primers directed towards conserved sequences in the tyrosine kinase domain (Figure 1). This strategy has proved to be successful before to isolate a range of genes belonging to the RTK family (17,18). The expression of RTKs was examined using five different strategies. 1) To verify the expression in retina, specific primer Q RT PCR analysis was performed using total retina cDNA as template. 2) Specific primer Q RT PCR analysis was also performed using RGC-specific cDNA as template. 3) *In situ* hybridization analysis of retinal sections using specific probes was used to show that cells expressing the different RTK mRNA were present in the ganglion cell layer. 4) Immunohistochemical analysis of sections of retina containing fluorescently pre-labelled RGC was used for RTKs. 5) Expression analysis using Q RT-PCR of whole retina with specific primers for some of the less studied RTKs that were identified, as well as on two putative ligands.

## Material and methods

### Animals and surgical procedures

Female Sprague-Dawley rats (each weighing 225–250 g) were obtained from B&K Universal (Sollentuna, Sweden). For axotomy experiments female and male Dark Agouti rats each weighing 130–250 g were used (B&K Universal, Sollentuna, Sweden), sex and weight differing between groups and occasions of operation. For experimental manipulations, animals were anaesthetized with an intraperitoneal injection mixture of xylazine (10–15 mg/kg, Rompun; Bayer AG, Leverkusen, Germany) and ketamine (30–100 mg/kg, Ketalar; Parke-Davis, England). Rats were fed *ad libitum*, housed in standard cages in temperature-controlled rooms with a 12-hour light and 12-hour dark cycle, light intensity ranging from 8 to 24 lux. Experiments were carried out in accordance with European Community guidelines and Association for research in vision and ophthalmology for use of animals in ophthalmic and vision research. The local ethics committee for experimental animals (Uppsala Djurförsöksetiska nämnd) scrutinized the procedures. RGCs were retrogradely labelled from the superior colliculi using previously described techniques (6,19,20). In brief, after exposing the surface of the superior colliculi, a piece of gel foam soaked in 0.9% NaCl, 10% dimethylsulphoxide (Merck), and 3% FluoroGold (Fluorochrome Inc., Englewood, Colorado, USA) or True Blue (Molecular Probes, Leiden, Netherlands) was applied onto these structures. Rats were sacrificed 7 days after tracer application by an intraperitoneal injection of 3–4 mL pentobarbital (100 mg/mL).

For intra-orbital optic nerve transection an incision was made in the skin covering the superior orbital rim (6), the superior orbital contents were dissected, and superior and external muscles were sectioned. Following rotation of the eye, the dura mater sheath was longitudinally opened and the optic nerve was completely transected as close as possible to the eye. Care was taken to avoid damage to the retinal blood supply and the retinal perfusion after operation was confirmed using an ophthalmoscope. Animals were sacrificed 12 hours, 1, 3, 4, 7, or 14 days after the transection. Each group contained 4–8 animals. Optic nerve transection (ONT) was chosen since this type of injury affects mainly the RGCs and to a lesser extent the other cells in the retina.

### Isolation of RGCs, Q RT PCR, and cloning

Total retina cDNA was synthesized from mRNA prepared from dissected retina. RGCs were isolated, and single-cell cDNA synthesis was performed as previously described (16). In brief, separation of the GCL from the rest of the retina was performed on a glass slide after enzymatic treatment of the retina. Individual, FluoroGold-labelled RGCs were identified in fluorescence microscopy, aspirated using a micropipette, washed, and transferred to reaction tubes for reverse transcription of mRNA. For the RGC-specific cDNA used for verification purposes, more than ten isolated RGCs were pooled, and the cDNA from those cells was used as a template. Negative controls were cell cDNA reactions with reverse transcriptase omitted. The medium containing the cells after layer separation was analysed for extracellular mRNA contaminants using Q RT PCR with Gapdh primers (16). Positive controls were performed on single cells by using primers to β-actin, Gapdh, Ret, Met, ErbB2, and TrkB (16). The conserved amino acid sequences, without gaps, HRDLA(A/T)RN and DVWS(F/Y/L)G(V/I) (Figure 1), were reverse-translated to nucleotide sequences with nucleotide sequence degeneracies of 6.5 × 10^5^ and 1.6 × 10^4^, respectively. This is a high degree of degeneracy for primer pairs (1010), but it has been shown to give effective amplification (21). The concentration of each specific primer with this degeneracy is very low, and inosines can be introduced to lower degeneracy, thus increasing the concentration of the specific primers in the ‘primer mix’. We used an empirical approach to find optimal primers and conditions (see below) for amplification which gave the following primer sequences that contain inosines: 5´-CA(C/T)(A/C)GIGA(C/T)(C/T)TIGCI(G/A)CI(C/A)GIAA-3´ and 5´-A(C/T)ICCI(A/T)(A/G)I(C/G)(A/T)CCAIAC(A/G)TC-3´. The degeneracy of the primers including the inosines is 64 for both primers. Inosine is a purine that can form base pairs with the other bases, cytidine, thymidine, and adenosine, but to a lesser degree with guanosine. The approach using multiple pools of primers in order to decrease primer degeneracy was disqualified by the limited amount of cDNA template for each reaction. The programs CODEHOP (22,23) and HYDEN (21) facilitate primer design. The typical PCR reaction contained 60 pmol of degenerate primers, 15 μL of single-cell cDNA reaction, buffer (15 mM Tris-HCl, 50 mM KCl), 0.2 mM of each of the deoxyG,A,T,CTP (dNTP), 2.5 mM MgCl_2_, and 2.5 U Amplitaq Gold DNA polymerase (Perkin-Elmer, Boston, MA, USA). DNA polymerase was activated (95°C for 10 min) followed by 2 cycles (94°C for 1 min, 37°C for 5 min, 65°C for 3 min) and 48 cycles (94°C for 1 min, 53.4°C for 5 min, 65°C for 3 min) using slow ramping (1°C/sec) between annealing and extension steps. PCR reactions were analysed by 1.8%–2.2% agarose gel electrophoresis, and PCR fragments of correct size were excised under ultra violet (UV)-light and extracted using silica-based chemistry (Qiagen, Hilden, Germany). Extracted PCR fragments were cloned in pMOS*Blue* vector using pMOS*Blue* blunt end cloning kit (Amersham, Buckinghamshire, UK). Clones were sequenced, and homology searches were done using NCBI BLAST against translated GenBank/Swiss Prot databases, and ClustalW was used for alignments. A positive identification required an identical match to a rat protein sequence or a match on an orthologous sequence with an E-value <10–30. Partial sequences were disregarded. For specific PCR primers, see Table I.

**Table I.** PCR primer sequences.

Target gene	Forward primer	Reverse primer
Axl	GTGTTCCTGCCCACTCAGAT	GCTAGGTCCCGGTGTATGAA
Csf-1R	GTGGCTGTGAAGATGCTCAA	GTTCTCATGCTGTCCCAGGT
Jak1	GCGACATTCTCCAAAGAAGC	TGCGCAAACAGATACTCCAG
Jak2	ACCCAAGTGGACAGAGTTGG	GCTGTTCAGATCACGGATGA
Met	TCCACAACAAAACGGGTGCG	GCACACCGAAGGACCACACGT
Non-sense	TCTATAGCATGAGCTACGATG	GCCAGTGACGTAGCATGTCTA
Pdgfrβ	GAGAGCGACGGTGGTTACAT	GACTCGATGTCCGCGTATTT
Vegfr-2	AAGCAAATGCTCAGCAGGAT	GAGGTAGGCAGGGAGAGTCC
Vegfr-3	GGACTAAGGCTCCAGGTTCC	CCCGCTGTCTGTTTGGTTAT
Ret	AAGAAAACGCCTCCCAGAGT	CAAGCCCCGTACAACTTGAT
Sky	TCAGGCCTTATCCTGGAATG	CTGGGTTCCATTTTCTTGGA
Rhodopsin	CCTGCTCAACTTGGCTGTGGC	GCCTGTGGGCCCAAAGACAA
Ros	TGCTGAATGTACCCAAGCTG	CCACTGAGGGTTCTGACCAT
TrkB	CCCCCAGTACTTCGGTATCA	TTCTCCAAGCTCCCTCTTCA
TrkC	ACAAGATGCTTGTGGCAGTG	CATGCTGCAGGTTAGTGAGC

### In situ hybridization histochemistry

Rat retinas were sectioned (12 μm) using a cryostat, and sections were mounted on poly-D-lysine-coated slides. Oligonucleotides complementary to the available rat receptor mRNAs identified in the PCR analysis were used as probes (Table II). Controls were performed by using a non-sense probe or by adding 100 times excess of unlabelled probe in order to compete out the labelled probe. Oligonucleotides were labelled with ^35^S-ATP (NEN, Perkin Elmer, Boston MA) using terminal deoxynucleotidyl transferase (Amersham, Buckinghamshire, UK) and purified on ProbeQuant G-50 columns. ^35^S-labelled oligonucleotides (10^6^ counts per minute/slide) in hybridization solution were hybridized to sections in a humidified chamber at 42°C for 14–16 hours. Slides were washed in sodium saline citrate (SSC) at 56°C, rinsed in water, dehydrated, dipped in NTB2 Kodak photoemulsion, and exposed for 4–6 weeks. Slides were developed in D19 developer (Amersham, Buckinghamshire, UK), counterstained with cresyl violet, and viewed and documented in a Zeiss Axioplan2 microscope.

**Table II.** Oligonucleotide *in situ* probe sequences.

Target gene	In situ probes
Axl	TGTTCTCATTCAGCATGCAGTTCCGCGCCGCCAAATCCCTGTGCACCA
Csf-1R	GCGTTTCTTGTGGTCAGGGTGCTTCCGGGAGATTCAGGGTCCAAGGTC
Met	GATCTGTCTGCCAGCAGTTTATAAGGTTTTCCCCACTAGTGCACCCCT
Pdgfrα	GCCCCACACTGAAGGTTCCGTTGAAGCCCTGCCTGCTGTCGTAGGAGG
Pdgfrβ	GCGTCACCTCCAGCTGGGGGTCTGTCACTCGGCATGGAATCGTCGTCT
Ros	TACCCCATTTCATCATCCGCTGTGTACCCCACTGTGCTGACGGCTCCC
Sky	GGAGCTGCAGGCGGTGCTTGAAGGCGAATAATGGCTGGTCGGGAAGTG
TrkB	CACAGACACCGTAGAACTTGACAATGTGCTCGTGCTGGAGGTTGGTCAG
TrkC	GTCCTCCCACCCTGTAGTAATCAGTACTGTAGACGTCCCTGGACATGCC
Vegfr-2	ATGCCATGCTCGTCACTGACAGAGGCGATGAATGGTGACCTGTGATCT
Vegfr-3	GCCTGCTTTCTATCTGCTCAAACTCTTCTGAGGCCAGCACCATCCCGC

### Immunohistochemistry

Rats were anaesthetized and perfused with 4% paraformaldehyde (PFA) in phosphate buffered saline (PBS). Eyes were postfixed, cryoprotected, sectioned (12 μm) using a cryostat, and mounted on Superfrost Plus slides. Sections were pre-incubated in 0.3% Triton X-100 in PBS (PBT) and incubated overnight with primary antibodies: rabbit α-mouse Csf-1R (1:200; #06-174, UpState), mouse or rabbit α-mouse Vegfr-2 (1:25, CH-11; 1:500, #07-158, UpState), rabbit α-mouse Pdgfrα,β (1:50, provided by L. Rönnstrand, Ludwig Institute for Cancer Research, Uppsala branch, the antibody recognizes both α and β types), goat α-mouse Ret (1:50; R&D Systems), mouse (1:40; 75133) or rabbit α-TrkB (1:200; 1494 R&D Systems), goat α-human Sky (1:50; Santa Cruz Biotech) in PBT with 5% of appropriate serum(s). Secondary antibodies were Alexa Fluor 488/594 conjugated donkey α-rabbit (1:50; Molecular Probes); Alexa Fluor 488/594 conjugated chicken α-goat (1:50; Molecular Probes), and Alexa Fluor 488/594 conjugated goat α-mouse (1:50; Molecular Probes). PDGF-α,βR was detected using biotinylated anti-rabbit (1:30; Vector) and a Vectastain ABC Elite kit (Vector Laboratories, Burlingame, CA, USA) with diaminobenzidine substrate. Negative controls were performed by omitting the primary antibody. Slides were mounted with Vecta-Shield (Vector Laboratories, Burlingame, CA, USA), and viewed and documented with a Zeiss Axioplan2 microscope. Cell counting in at least three retinas was performed in the central regions of the retina. Only cells with clear True Blue labelling were counted in ten visual fields (40× objective) from each retina. Percentages were calculated in relation to the total number of clearly True Blue-labelled cells in the ganglion cell layer.

### Quantitative Q RT PCR

Total RNA was prepared from whole retinas using Trizol (Invitrogen). The RNA was analysed for purity with Agilent RNA 6000 Assay (Agilent Technologies, USA). After treatment with RNase-free DNase (Promega, Madison, WI), 1 μg of total RNA was used for cDNA synthesis with TaqMan Reverse Transcriptase reagents using random hexamer primers (PE Applied Biosystems). Analysis of mRNA levels was performed using SYBR Green I assay in combination with ABI PRISM 7700 Sequence Detection System (PE Applied Biosystems). Sequence-specific primers were designed using Primer Express software (Applied Biosystems), for sequences see Table III. PCR reactions were carried out on duplicate cDNA samples in a 96-well plate using final concentrations of 1× SYBR Green PCR Master Mix (Applied Biosystems) and 300 nM of forward and reverse primers respectively (Table III). The PCR conditions were: 95ºC for 10 min, followed by 40 cycles of: 95ºC for 15 s, 60ºC for 60 s. Specific amplification was confirmed by generating dissociation curves and separation by agarose gel (3%) electrophoresis. For each sample, a C_T_ (threshold cycle) value corresponding to the PCR cycle at which the fluorescent signal reaches a threshold above the base-line emission was determined. Based on this, initial target mRNA levels were calculated, and relative differences between operated (subjected optic nerve transection) and unoperated animals were determined. As controls, the relative mRNA levels of the housekeeping genes β-actin and Gapdh were measured in each group of normal and axotomized tissues. The control genes did not show differences in the mRNA levels between the groups and thus were not affected by optic nerve transection. β-Actin was chosen as reference gene, and all collected data were normalized to β-actin expression. Thy-1, an RGC-specific gene, was analysed in order to monitor changes of expression in RGCs. Statistical analyses were performed using analysis of variance (ANOVA). *P*-values less than 0.05 were considered significant.

**Table III.** Real time PCR primer sequences.

Target gene	Forward primer	Reverse primer
Axl	CAGGTACCGTGCCCGAAA	TCGCTGATGCCCAGACTGT
Sky	CCCGGTCTTTCAATCGAGAA	AATTCATCGCTGATGCCCA
Gas6	CCCGGACATCGCTGGAT	GAATGCGAGCCACGACTTCTA
ProteinS	AAAGGCAACTCGCCATCTTG	TGGAACGCCACCCAGGTA
Pdgfrβ	CCATCAACGTTACTGTGATCGAA	CTCAGCAATTTGTACGTCTTCCA
Vegfr-2	GCACCATGCAGACGCTGA	GCTGCCAGTACCATTGGATGT
Csf-1R	GCTCTGATGTCCTGCTCTGTGA	CCTGCACTCCATCCATGTCA
Thy-1	CAGCCTGCCTGGTGAACC	GGATGGGCAAGTTGGTGTTATT
β-Actin	CTTCAACACCCCAGCCATG	GTGGTACGACCAGAGGCATACA
Gapdh	AAGCTGGTCATCAATGGGAAAC	TCACCCCATTTGATGTTAGCG

## Results

### PCR controls

**Figure 2.**
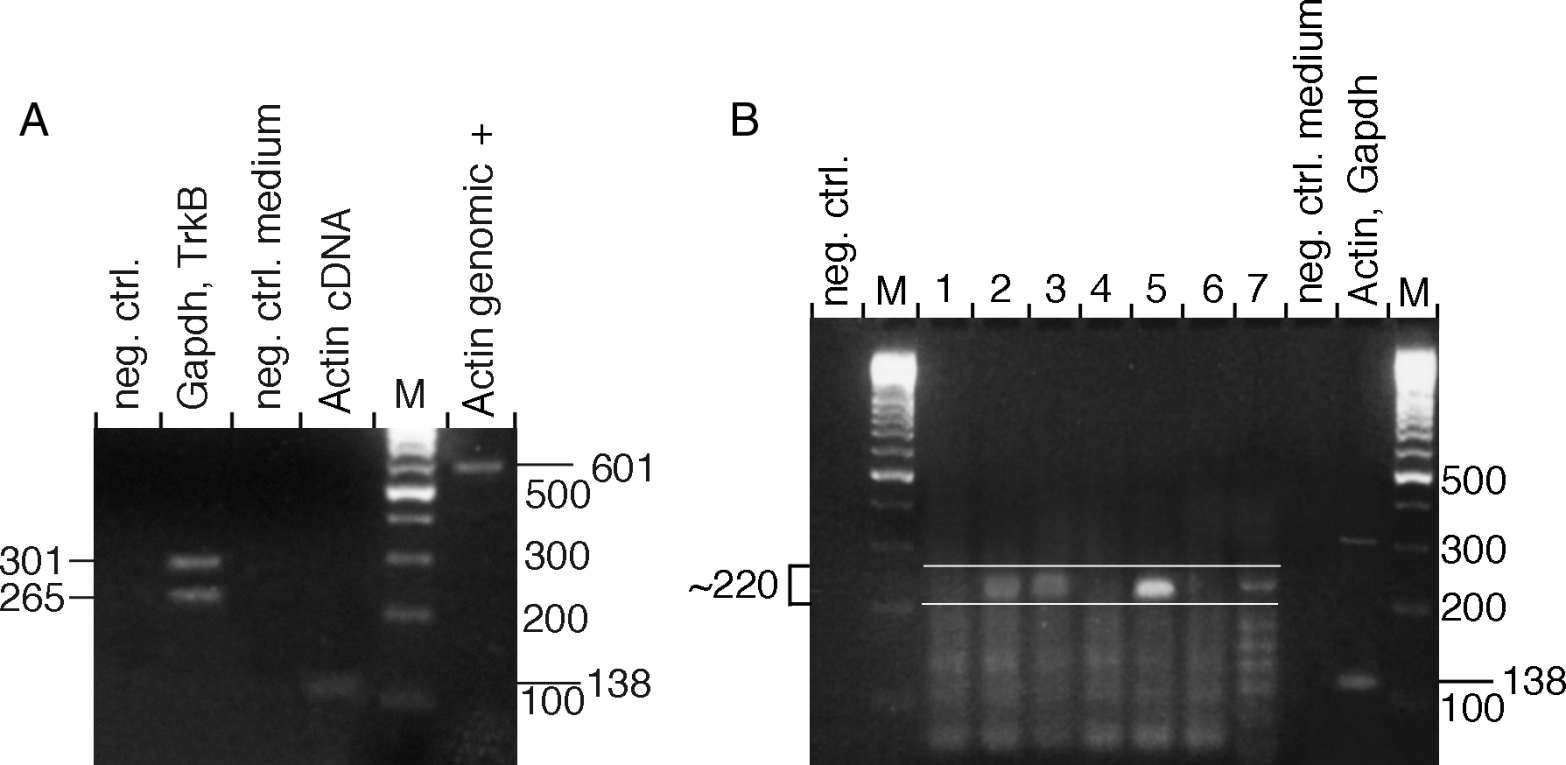
Agarose gel electrophoresis analysis of PCR products from single-cell degenerate primer PCR. A: Control analyses of the single-cell PCR showing negative and positive control reactions. (Lanes: neg. ctrl., PCR on cDNA reaction without reverse transcriptase; single retinal ganglion cell cDNA analysed using specific primers to Gapdh and TrkB; neg. ctrl. medium, control for extracellular RNA contamination; Actin cDNA and Actin genomic, controls for cDNA synthesis and genomic DNA contamination with primers that give a 138 bp fragment with the actin cDNA and 601 bp with genomic DNA as templates; M, 100 bp DNA size marker ladder). B: PCR on seven representative single cells using degenerate primers directed towards conserved sequences in the tyrosine kinase domain of receptor tyrosine kinases. (Lanes: negative controls and markers as in A; positive control using specific primers for Actin and Gapdh; 1–7, degenerate primer PCR on single retinal ganglion cell cDNA). Specific PCR products are approximately 220 bp. Cells 2, 3, 5, and 7 are considered positive, while 1, 4, and 6 are negative.

To identify RTKs expressed by RGCs we used degenerate primer PCR directed towards conserved sequences in the tyrosine kinase domain (Figure 1A). The PCR was applied on cDNA from acutely isolated adult RGCs. Both as controls and as part of the experiment, specific primers to several genes that are expressed by all cells (Gapdh, β-actin), in RGCs TrkB, Ret (24), as well as to genes not being expressed by RGCs (TrkA, rhodopsin), were included in the analysis (Table IV). Included were also Met and ErbB2 as candidate RTKs that we have previously found being expressed in RGCs (16). A pair of random primers was used as a negative control (non-sense). The specific primer PCRs were used to optimize and to control the procedure as also discussed elsewhere (16). In optimized standard reactions, primers spanning exon/intron boundaries in the actin gene did not produce any products. PCR using degenerate or specific primers on single-cell cDNA reactions without reverse transcription did not give any PCR products. Both controls confirm that the samples were free from genomic DNA contamination (Figure 2A). These kinds of controls were included in parallel to the analyses. The highly expressed housekeeping gene, Gapdh, could not be amplified from the medium in which cells were aspirated after the retinal layer separation (see Figure 1B and C), confirming that the surrounding solutions were free from mRNA contaminants.

**Table IV.** Summary of receptor tyrosine kinase (RTK) expression in adult rat retina and retinal ganglion cells (RGCs).

Amplified genes:	Frequency (%) / Cell distribution of amplified RTKs using the degenerate PCR^a^	Verified expression: Specific primer PCR on total retina cDNA	Verified expression: In ganglion cell layer using in situ hybridization (ISH) or immunochemistry analysis (IHC)	Verified expression: Specific primer PCR on RGC-specific^h^ cDNA	Verified expression: Identified^b^ RGCs (Fraction of identified RGCs ± SD %)
Identified RTKs					
Axl	12/2,3,8	+	+/-	+	nd
Csf-1R	9/1–3,8–10	+	+	+	+ (66 ± 16)
Eph A4^d^	2/6,7	nd	nd	nd	nd
Pdgfrβ	6/4,5,10	+	+^f^	nd	+^f^ (79 ± 16)
Ptk7^d^	3/8	nd^e^	nd^e,g^	nd^e^	nd^g^
Ret	3/9	+	+	+	+ (16 ± 15)
Ros	4/3,6,7	+/-	-	nd	nd
Sky	24/1,3,10	+	+	+	+ (18 ± 15)
TrkB	2/10	+	+	+	+ (62 ± 12)
TrkC	3/5	+	+	+	nd^g^
Vegfr-2	10/1,4,9,10	+	+	nd	+ (51 ± 25)
Vegfr-3	5/3,10	+	-	nd	nd
Cytoplasmic TKs					
Jak1	14/9	+	nd	nd	nd
Jak2	3/1,2,4,5	+	nd	nd	nd
Control genes					
β-Actin^c^	na	+	nd	+	nd
Gapdh^c^	na	+	nd	+	nd
Rhodopsin^c^	na	+	nd	-	nd
ErbB2^c^	na	+	nd	+	nd
Met^c^	na	+	+	+	nd
Pdgfrα^c^	na	+	+	nd	+^f^
Ret^c,i^	na	+	+	+	+ (16 ± 15)
TrkA^c^	na	-	-	-	nd
TrkB^c,i^	na	+	+	+	+ (62 ± 12)
Non-sense	na	-	na	-	na

^a^Frequency (%) / Distribution of RTK among the 10 positive picked single RGCs. ^b^Identification of retinal ganglion cells by retrograde filling using fluorescent dye from superior colliculus. ^c^Control genes/receptors included in the verification. Amplified using specific primers. ^d^Similar to mouse and human sequences/no or partial rat sequence available. ^e^The rat Ptk7 sequence is not available, primers could not be made. ^f^Pdgfr antibody recognizes both α and β receptors. ^g^Antibody not available or good. ^h^cDNA from pool of more than 10 picked RGCs. ^i^Control genes that were also identified using the degenerate PCR strategy na = not applicable; nd = not determined; - = below detection; +/- = weak signal; + = clear signal.

The success rate in the PCR analysis, as defined by cells that gave amplification with the Gapdh primers and were blank in the controls, was on average 82% but varied from 100% to 48% between experiments (cell-picking occasions). Using specific primers for Gapdh, TrkB, and Ret, 56% of the cells were positive for Gapdh and TrkB mRNA, and 20% of the cells were positive for Gapdh and Ret mRNA.

### Isolation and identification of cDNA fragments from RGCs encoding the tyrosine kinase domain

More than 40 cells were used for optimization of the degenerate primer PCR protocol, and typically 45% of these degenerate primer PCR amplifications gave products. This was lower than when using the specific primers. Optimized PCR was then carried out on 20 RGCs, and 10 cells gave clear PCR products of the predicted sizes. Seven such amplifications are shown in Figure 2B. The PCR fragments of the expected sizes (Figure 2B) were cloned and sequenced. Using the NCBI BLAST search against available databases with the PCR sequences as query, 25.4% of the sequences (110) were identical to 12 RTK and 2 cytoplasmic tyrosine kinases (E-values <10^-30^, orthologues excluded); Axl (‘anexelekto’/UFO/Ark/Tyro7, Q8VI99, AB067527), Csf-1R (colony stimulating factor-1 receptor, Q00495), Eph A4 (highly similar to mouse Eph A4), Pdgfrβ (platelet-derived growth factor receptor β, Q05030), Ptk7 (protein tyrosine kinase 7, highly similar to mouse Ptk7, AAH27800), Ret (CAC10584), Ros (AAA40968), Sky (Tyro3/Etk2/Dtk/Brt/Rse/Tif, P55146), TrkB (NP 036863), TrkC (Q03351), Vegfr-2 (Flk1/Kdr, NP 037194), Vegfr-3 (Flt4, AAL13269), and Jak1, 2 (XP 342873, NP 113702), while the rest (74.6%) only displayed partial, low, or no similarity at all (E-values >10^-20^) to the tyrosine kinase domain sequence. The number of tyrosine kinase sequences found using the degenerate PCR primer amplification from each individual cell varied substantially, ranging from only one tyrosine kinase sequence in several copies up to ten of the identified sequences among clones that were derived from a particular cell (Table IV, column 1). Furthermore, specific primers to TrkB gave PCR products in 68% of the cells that were positive for Gapdh, which is in agreement with histological analysis where a majority of the RGCs express TrkB (25). In contrast, only 2% of the cloned sequences corresponded to TrkB, and those came from just one cell out of the ten successful analyses.

### Verification of RTK expression in retina and RGCs

The expression of the identified RTK as well as additional genes were checked in retina using specific primer Q RT PCR analysis of cDNA from whole retina as well as from picked (Figure 1B and C) and pooled RGCs (RGC-specific cDNA from >10 RGCs). Rhodopsin could be amplified from total retina but not from the RGC-specific cDNA. TrkA could not be amplified from RGC cDNA. The other RTKs (TrkB, Ret, Met, ErbB2) could be amplified from both total retina and RGC-specific cDNA. The random sequence primers did not produce any amplification (Table IV).

**Figure 3.**
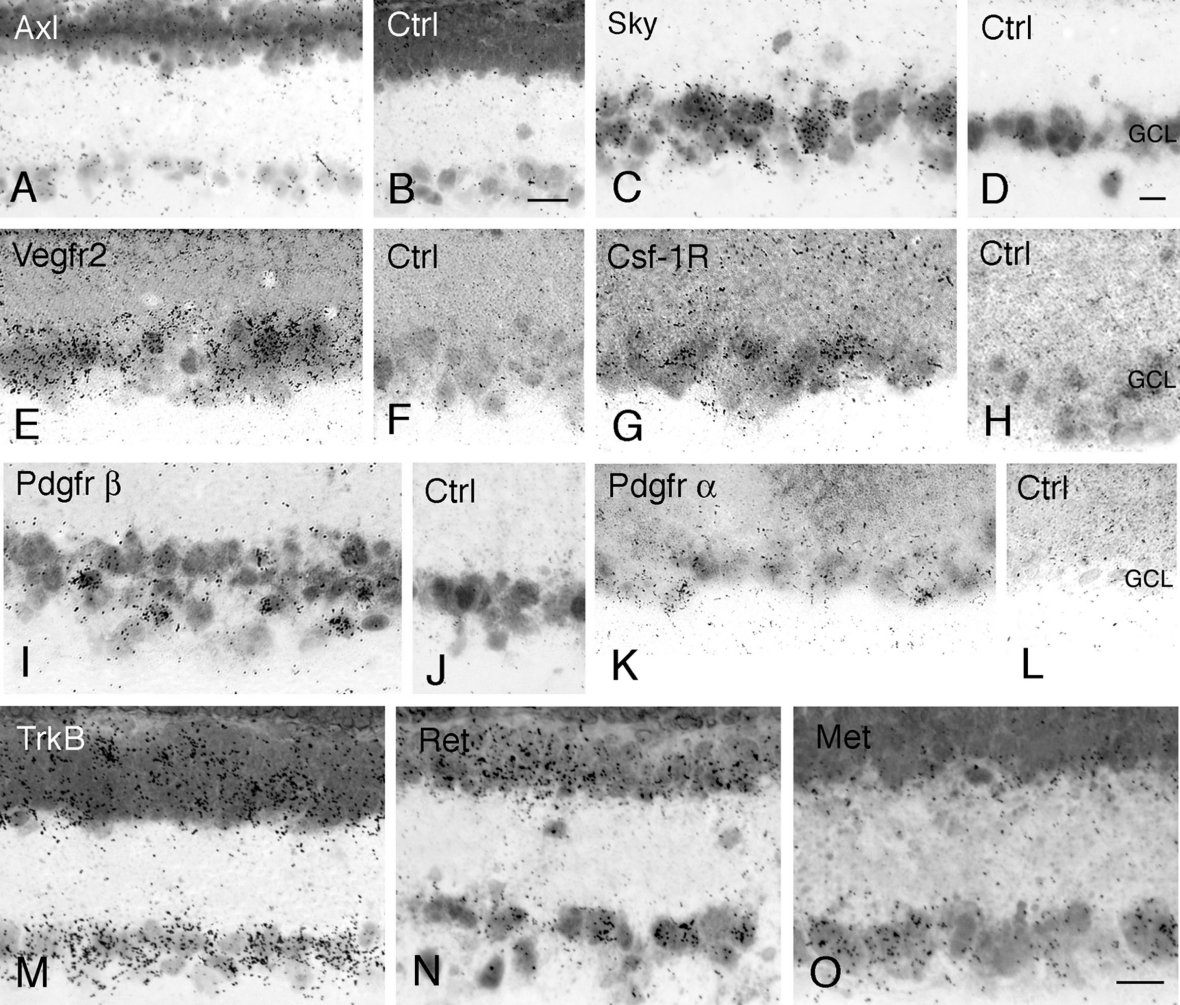
Expression of receptor tyrosine kinase (RTK) mRNA in the retinal ganglion cell layer. Bright-field micrographs of oligonucleotide probe *in situ* hybridization analysis of the retinal ganglion cell layer in adult rat. Autoradiography for Axl (A), Sky (C), Vegfr-2 (E), Csf-1R (G), Pdgfrβ (I), Pdgfrα (K), TrkB (M), Ret (N), and Met (O) mRNA expression. B, D, F, H, J, and L: negative controls using 100 times molar excess of unlabelled oligonucleotides (GCL = ganglion cell layer). Scale bars 20 μm. Bar in B also valid for A, bar in D also valid for C–L, and bar in O also valid for M–N.

Among the 12 identified RTKs, rat sequences for Ptk7 and Eph A4 were not available, and their expression in retina was not verified. The expression and main function of Eph A4 in retina and RGCs are well documented (26,27), and this gene was not further analysed. The RTK Ros was identified using the Q RT PCR strategy as being expressed in RGCs, but its expression could neither be conclusively verified in retina using Q RT PCR nor using histological methods. Axl and Vegfr-3 mRNA expression could be verified using PCR but the histological methods did not give confirmation of these results (Table IV and Figure 3A).

Axl and Sky/Tyro-3 belong to the same subfamily of RTKs. In contrast to Axl, labelling for the Sky was clear in retina and retinal ganglion cell layer using the different methods (Table IV, Figure 3C and 5B). Displaced amacrine cells constitute up to 50% of the cells in the ganglion cell layer, and to confirm the RGC localization of the identified receptors we studied whether Sky immunoreactivity was present on RGCs retrogradely traced with True Blue. As shown in Figure 5A–C, Sky is expressed by RGCs as its immunoreactivity co-localizes with True Blue-positive RGCs (Figure 5A–C).

**Figure 4.**
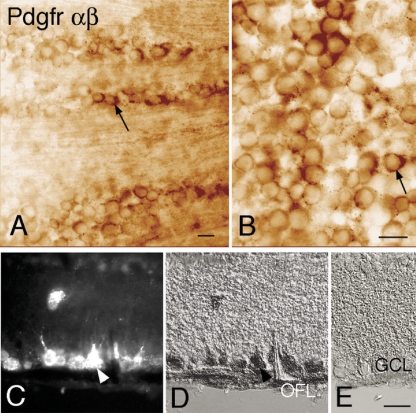
Immunohistochemical localization of Pdgf receptors in the retinal ganglion cell layer. Immunohistochemistry using an antibody against both α and β forms of the Pdgf receptor. A, B, and D: Bright-field micrographs of adult rat retina with immunoreactivity visualized using diaminobenzidine precipitates. A, B: Micrographs of the vitreal side of whole-mount retina preparation with the focus plane in the ganglion cell layer. Pdgfrα,β immunoreactive cell (arrow). Immunoreactivity could also be seen in the optic fibre layer (out of focus in panels A and B but visible in cross-section shown in D). C–E: Cross-sections of retina with FluoroGold-labelled RGCs. C: Fluorescence micrograph showing FluoroGold-labelled RGCs (arrow-head). D: Pdgfrα,β immunoreactivity in the same section as shown in C. Note the displaced RGC in C, which is also labelled for Pdgfrα,β. E: Negative control (GCL = ganglion cell layer; OFL = nerve fibre layer; RGCs = retinal ganglion cells). Scale bar in A, B, and E, 20 μm, also valid for C and D.

**Figure 5.**
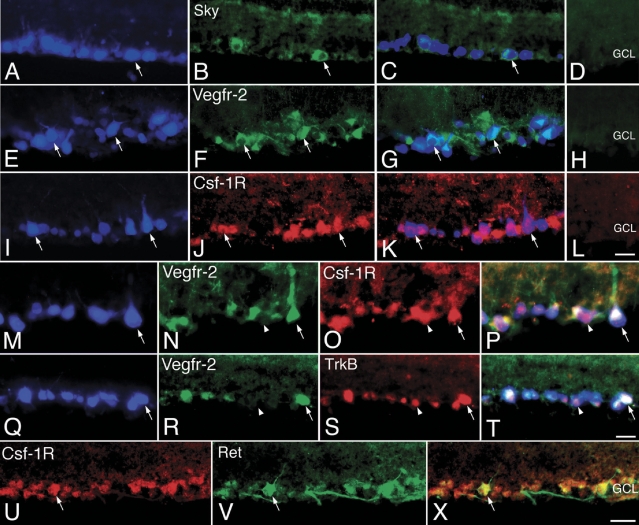
Immunohistochemical localization of receptor tyrosine kinases (RTKs) in retrogradely filled retinal ganglion cells. Adult rat retinal ganglion cells (RGCs) were retrogradely filled with True Blue from the superior colliculi, and cross-sections of the retinas were subjected to epifluorescence immunohistochemistry. A, E, I, M, and Q: Micrographs showing RGCs with True Blue fluorescence (blue). B: Sky; F, N, and R: Vegfr-2; V: Ret immunoreactivity (green). J, O, and U: Csf-1R; and S: TrkB immunoreactivity (red). C, G, K, P, T, and X: Merge of panels to the left on same row. Arrows indicate labelled (A–K) and double-labelled (M–X) True Blue-identified RGCs. Arrow-heads indicate single-labelled RGCs. D, H, and L: Negative controls (GCL = ganglion cell layer). Scale bars in L and T are 25 μm and are also valid for A–T. Scale bar in X is 40 μm, also valid for U and V.

**Figure 6.**
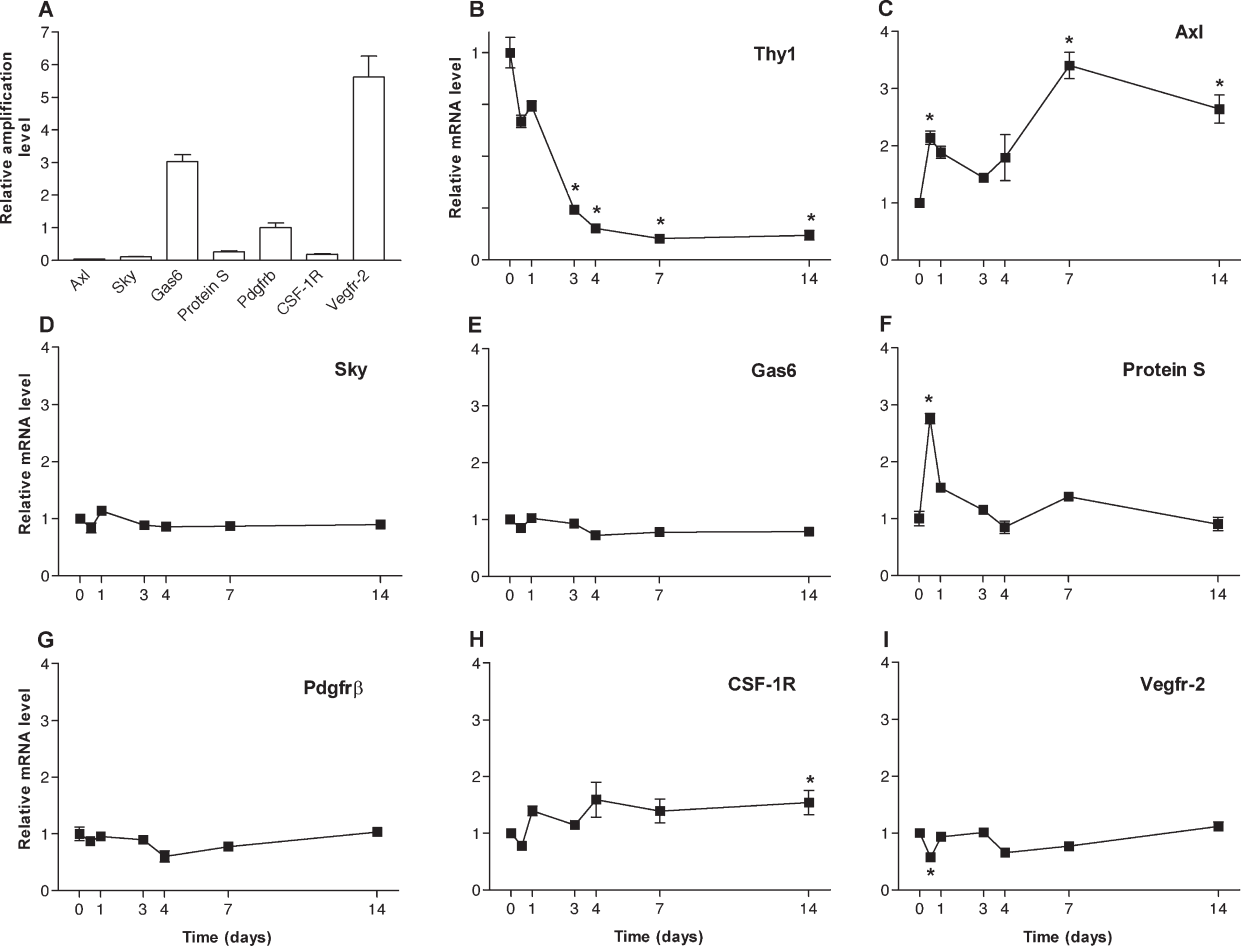
Relative mRNA levels in normal retina and retina from rats 12 hours, 1, 3, 4, 7, and 14 days after optic nerve transection. Relative mRNA levels were analysed using quantitative real-time PCR analysis. A: Relative amplification levels in normal retina. Relative mRNA levels for Thy-1 (B), Axl (C), Sky (D), Gas6 (E), ProteinS (F), Pdgfrβ (G), Csf-1R (H), and Vegfr-2 (I). Time point 0 represents normal levels and was assigned the value of 1. *n* = 6. Error bars show SEM. * indicates *P* <0.05 ANOVA.

Labelling for Vegfr-2 mRNA was found in the GCL (Figure 3E, F) and in the inner nuclear layer (data not shown). Vegfr-2 immunoreactivity was seen on True Blue-labelled RGCs in the GCL (Figure 5E–H). Weak labelling for Csf-1R mRNA was found in the GCL (Figure 3G, H) and Csf-1R-immunoreactivity was seen on True Blue-labelled RGCs (Figure 6I–K). Pdgfrβ was identified using the PCR strategy, and labelling for Pdgfrβ mRNA was found over cells in the GCL (Figure 3I, J). Labelling for Pdgfrα mRNA could also be detected (Figure 3K, L), and using an antibody that detects both Pdgfr α and β forms immunore activity was detected on cells in the GCL. Whole-mount Pdgfrαβ immunohistochemistry labelled RGCs in the GCL as well as axons in the optic fibre layer (Figure 4A, B). This result was confirmed by Pdgfr immunoreactivity in the GCL on FluoroGold-labelled RGCs (Figure 4C–E).

### RGCs express multiple RTKs

It was clear from the PCR and histochemical analyses that several of the analysed RTKs were expressed in RGCs. Not all RGCs were labelled, suggesting that there are subpopulations of RGCs that express the receptors. Counting RTK-immunoreactive True Blue-labelled cells in relation to the total number of True Blue-labelled cells, in the GCL of the central part of the retina, allowed the percentage of RGCs to be calculated for Sky, Vegfr-2, Csf-1R, Pdgfrαβ, TrkB, and Ret. The results are shown in Table IV (rightmost column) and Figure 5. The calculation is based on the fact that by applying FluoroGold to both superior colliculi 98.4% of the RGCs present in the adult rat retina are traced (6).

Co-labelling for RTKs in RGCs was analysed using double immunohistochemistry. Retinas with True Blue-labelled RGCs were used, and we found that Vegfr-2 and Csf-1R immunoreactivity (Figure 5M–P) overlapped in 49% ± 24% of the True Blue-labelled cells; 14% ± 12% of the cells were positive for Csf-1R and not for Vegfr-2 (Figure 5O, P). Very few cells that were labelled for Vegfr-2 and not for Csf-1R could be seen. Vegfr-2 immunoreactivity overlapped with that of TrkB in 46% ± 20% of the True Blue-labelled cells (Figure 5Q–T); 12% ± 11% of the cells were positive for TrkB and not for Vegfr-2. Just a few cells were labelled for Vegfr-2 and not for TrkB. The clear majority of Sky-labelled cells were also labelled for Vegfr-2 (data not shown). The analysis of the overlap of TrkB and Csf-1R was not conclusive due to uncertain staining but indicated that the patterns overlap (data not shown). The patterns of Csf-1R and Ret overlapped in the GCL (Figure 5U–X), and the TrkB labelling overlapped with those of Sky and Ret (data not shown) (24). A potential overlap between Ret and Sky patterns could not be analysed because both antisera were raised in goat, and Pdgfr immunoreactivity could not be analysed because the double labelling epifluorescence analysis was not conclusive.

### Expression in retina after optic nerve transection (ONT)

Little is known about the roles for several of the above genes in the retina. So next we studied whether ONT induces changes on their mRNA expression level at 12 h, 1, 3, 4, 7, and 14 days after ONT. This analysis was done by using Q RT-PCR. Basal levels of Pdgfrβ, Csf-1R, Vegfr-2, Sky, and Axl, as well as two ligands for Axl/Sky, Gas6 and ProteinS, in normal and injured adult rat retina were analysed (Figure 6A). Levels of mRNA vary between the genes in normal retina, with high levels in retina of Vegfr-2, Gas6, and Pdgfrβ mRNA compared to Axl and Sky. The levels shown in Figure 6A should not be considered as absolute relative mRNA levels due to possible variances in amplification efficiency between primer pairs. However, our experience is that differences in amplification levels reflect the differences in mRNA levels. The expression levels were normalized using β-actin mRNA levels. β-Actin and Gapdh mRNA levels were 22 and 117 times higher, respectively, than that of Pdgfrβ in retina (data not shown). Thy-1 mRNA levels were analysed to visualize RGC-specific expression in relation to RGC loss. There was a marked decrease of Thy-1 mRNA levels that parallels the loss of RGCs after ONT (Figure 6B). The levels of Axl (Figure 6D) and ProteinS (Figure 6F) mRNA increased after ONT, while the mRNA levels of the other analysed genes remained in general unaltered compared to normal levels (Figure 6C–I).

## Discussion

The reason for undertaking this study was to get a better picture of the repertoire of RTKs in RGCs. We have used a degenerate primer PCR-based method and have amplified RTK sequences from cDNA from single RGCs. Twelve RTKs were identified (Table IV) of which several have previously not been primarily associated with RGC function. Using different methods, we have been able to confirm their expression in adult RGCs. The results also suggest that multiple RTKs are expressed by the same RGC and that there are subpopulations of RGCs that express different receptors.

Detecting specific transcripts in a single neuron is a major technical challenge, owing to the very small amounts of mRNA involved. The most straightforward approach is to analyse the expression of a single gene using a pair of specific primers (28), and, if several genes are to be analysed, to split the cellular content in several reactions. However, splitting the cellular content will decrease the sensitivity of the technique, which is already close to its limits. Gene-specific pre-amplification ‘multiplex PCR’ of the different genes before splitting into several reactions (16,29) compensates for the drop in sensitivity. An alternative is a combination of the single-cell technique with degenerate primer PCR, which was used in this study, and which has been used to characterize ion channels in interneurons (28,30). Any approach to characterize single-cell mRNA using PCR suffers from serious limitations, and single-cell genetic approaches face the problems of false negative genes (genes expressed but not detected in sample). It should be emphasized that those approaches are generally not useful for quantification of the amounts of cellular mRNA.

We obtained unspecific amplification in the degenerate PCR (75% of all sequences). This was most likely due to the high degree of degeneracy of the target sequences. The primer set was a result of an empiric approach where the target amino acid sequence, primer degeneracy, primer concentration, and not least the limited amount of template, were taken into consideration. The introduction of inosines in the primer sequence, resulting in lower primer complexity (64) and a higher effective concentration of each specific primer, was important for a successful amplification of the limited amount of template but resulted in a higher degree of unspecific amplification. This trade-off may also explain the skew seen between the amplification results and the verified expression in identified RGCs. This is most obvious for TrkB and Sky. TrkB is expressed in about two-thirds of the RGCs but was only found a few times among the amplified sequences. Sky immunoreactivity was found on less than a fifth of the identified RGCs (Table IV) but was the sequence most often amplified. Inosine in the primers is not fully neutral in its base-pairing to the four bases and may then contribute to a skewed amplification. However, comparing the sequences of rat TrkB and Sky at the positions of the inosines, there are no obvious differences, such as more guanulate nucleotides in the TrkB than in the Sky sequence, that explain the uneven amplification levels. Another variable that can give rise to a skewed result is differences in RTK mRNA expression levels. A low mRNA expression may be below the detection level of the PCR. Ros was identified using Q RT PCR; however, the retinal expression could not be firmly confirmed by the *in situ* hybridization. This indicates that Ros sequences were amplified in spite of low mRNA levels in RGCs and retina. This argues against the mRNA level being the critically limiting factor for amplification. The distribution of RTKs among the sampled cells (Table IV) was overall in agreement with the results obtained by the immunohistochemical analysis, with the exception of Ros. These results confirm that it is hazardous to draw conclusions about expression levels based on single-cell PCR, particularly using degenerate primers. This together with the limited success rate (only 10 out of 20 cells gave an amplification product) made us not to consider the amplified sequences, derived from the single-cell degenerate primer amplifications, as being decisive for determining the repertoire of RTKs in RGCs but rather being indicative and a subject for further investigations.

We used specific primers directed to several genes that are expressed in the retina as well as in RGCs (Table IV). TrkB and Ret but also ErbB2 and Met were analysed as RTK candidates that should be present in RGCs, and the expression of these genes could be detected both in total retina as well as in the RGC pool. Rhodopsin is expressed in photoreceptors and not in RGCs, and Gapdh as well as β-actin are genes that are expressed in all cells. TrkA should not be expressed in rodent RGCs. The results from the present study are in agreement with those presumptions. Jak1 and 2 are cytoplasmic tyrosine kinases that were picked up by the degenerate primers. Both Jak1 and 2 mRNA and protein have been found in the murine adult GCL (31). The primers were not designed to exclude cytoplasmic tyrosine kinases; however, the cytoplasmic kinases diverge at a higher degree in the amplified region from the RTKs (2), and this may explain why few cytoplasmic kinase sequences were amplified.

The identified RTKs belong to several subfamilies. The largest subfamily hosts the Eph receptors, and a representative, Eph A4, for this family was found. Eph A4 is expressed by developing and adult RGCs, and its function is in axon guidance and boundary recognition (26,27). Axl (also known as UFO, Ark, or Tyro7) (32) and Sky (Tyro3, Etk2, Dtk, Brt, Rse, or Tif) (33) constitute together with Mer (34) a RTK subfamily. Sky and Axl but not Mer were amplified from single RGCs. However, only Sky expression could be conclusively verified using PCR as well as using *in situ* and immunohistochemistry in RGCs. Q RT-PCR of Sky showed relatively low mRNA levels in normal retina, and the levels were not altered remarkably after ONT. ONT is an injury that mainly affects the RGCs since it essentially is an axotomy of the RGCs. Axl gave low signals in the PCR, showed low levels in the Q RT-PCR analysis, and could not be detected in the GCL using *in situ* hybridization, which shows that its expression is very low. Although the mRNA levels of Axl were low in normal retina, they were up-regulated substantially after injury. Axl, Sky, and Mer are activated by the product of the growth arrest-specific gene 6 (Gas6) (35,36) that is abundantly expressed in the central nervous system (CNS) (37). Gas6 is anti-apoptotic for CNS neurons (38), and its expression is regulated in sciatic nerve after nerve transection (39). The Q RT-PCR showed a relatively high level of Gas6 in normal retina, but the alteration after optic nerve transection was minor, and an injury-induced regulation is therefore less likely. ProteinS is structurally related to Gas6 and may function as a ligand to Sky and Axl (40,41). ProteinS mRNA levels increased acutely to approximately three times the basal level 6 hours after ONT. Axl has been shown to regulate endothelial cell activation by negative feedback exerted on Vegfr-2 signalling (42). The same receptor ligand systems are expressed in RGCs and similar exerted effects on signalling are likely during interactions between RGC axons, astrocytes, and maybe blood vessel cells. In addition, the third member, Mer, is expressed by photoreceptors, and progressive loss of photoreceptors is seen in animals with targeted deletion of the Mer gene (43) or in animals with a natural null mutation as in the RCS-rat (44).

The PCR identified Vegfr-2 (Flk1, Kdr) (45) and Vegfr-3 (Flt4) (46) in RGCs. Our results showed that both genes are expressed in the retina, but only Vegfr-2 expression could be verified in RGCs (Table IV). Previous results show that Vegfr-2 mRNA and protein (47,48) as well as Vegfr-3 immunoreactivity are present in the GCL (49). Q RT-PCR showed a high basal level of Vegfr-2. The regulation after injury was not significantly altered from normal levels except at 6 hours after injury, when there was a decrease. Vegf, the ligand for Vegfr-2 and -3, is known as a mediator of neo-vascularization, and more recently it has been shown to have neurotrophic properties on CNS neurons. Vegf promoted survival of mesencephalic neurons (50) and hippocampal neurons in culture (51) as well as promoted neurite outgrowth from retinal explants (48). We found that immunoreactivity of Vegfr-2 was co-localized with the Sky-expressing RGCs as well as with TrkB in approximately half of the RGCs. This points to complementary roles for Vegfr-2, Sky, and TrkB in RGCs.

Csf-1R is expressed by two-thirds of the adult RGCs (Table IV, Figure 3G, Figure 5J, O, U). Data from the Q RT-PCR showed a relatively low expression of Csf-1R in normal retina. The alterations in mRNA expression levels after injury were small and could not be statistically verified. Csf-1R functions are mainly associated with the immune system regulation, but data support functions in the nervous system. Csf-1R is expressed by microglia (52) but also by neurons (53,54). The ligand, Csf-1 (M-Csf), is mainly expressed by astrocytes (55) but has been found in neurons (56,57). Several effects on neurons including neuroprotective effects on cultured Purkinje cells (53) and hippocampal neurons (58) have been implicated. Mice lacking the Csf-1 gene had abnormalities in the developing nervous system (59,60) and showed increased neuron vulnerability to ischemic injury (61). The Pdgf receptors belong to the same RTK subfamily as Csf-1R. The α and β forms of the Pdgf receptors are expressed in neurons (62–64) and glia cells (65,66). The ligands have been shown to have neurotrophic activities (62,67). In retina, Pdgfrα is expressed by astrocytes in the optic nerve fibre layer and by large cells in the GCL (68). In agreement with those results, we found α and β mRNA labelling on cells in the GCL and immunoreactivity on a clear majority of postnatal RGCs (Table IV, Figure 4). Pdgfrβ mRNA levels were intermediate in normal retina, and the axonal injury did not cause any large alterations in their mRNA levels. A small decrease was seen at day 4 concomitant with the onset of RGC loss after axotomy. The Pdgf receptors are involved in a variety of auto- and paracrine cell interactions during development. Mice lacking the α-receptor die early with several embryonic abnormalities attributed to dysfunction of specialized cells such as sclerotome and neural crest cells. Mice lacking the β-receptor die just prior to birth and exhibit vascular, renal, and haematological defects. In retina, Pdgfrβ has been shown to be crucial for the development of microvasculature, but little is known about the functions in developing or postnatal retinal ganglion cells. Our results show that about half of all RGCs express Vegfr-2 and Csf-1R in addition to either Pdgfrα or β. As briefly exemplified, those receptors mediate interactions between glial cells, cells in the immune systems, and vascular cells. These results suggest that interactions with neurons and more specifically the RGCs, when it comes to interactions in the retina, should also be taken into consideration. In the adult retina, RGC axons, retinal vessels, and astrocytes may depend on a homeostatic relationship in which the present receptors and their ligands serve. The fact that several of the identified receptors are expressed on the same cells indicates that cross-talk between signal transduction pathways may also occur; this is exemplified by the negative feedback exerted by Axl on Vegfr-2 signalling in vascular endothelial cells (42).

When considering the extent of overlap between the studied receptors within the full RGC population, as suggested by the immunodetection results, it is possible to distinguish one group that consists of approximately half of the RGCs. This group contains RGCs that express TrkB, Csf-1R, Vegfr-2, and Pdgfrα/β. Within this group, subgroups of RGCs express Sky and Ret. The other half consists of subgroups of cells that either do not express any of the identified receptors or express TrkB and/or Csf-1R. This work shows that RGCs are heterogeneous in respect to which RTKs they express, and this work is a first attempt to use this feature to classify RGCs. Work remains in order to link any physiological function to the suggested RGC groups.

## Conclusions

Single-cell analysis is a powerful tool to analyse cell-specific gene expression but is also associated with several technical problems. The conclusion from this work is that the repertoire of receptor tyrosine kinases expressed by retinal ganglion cells is more extensive than has been suggested by previous work. The results also suggest that multiple RTKs are expressed by the same RGC and that there are subpopulations of RGCs that express different receptors. Several of the receptors found in this study (Axl, Sky, Pdgfrβ, Csf-1R, Vegfr-2) have not previously been primarily associated with retinal ganglion cell function. Alterations in mRNA levels after optic nerve transection suggest roles in an injury-induced system for Axl and ProteinS but not for Sky, Pdgfrβ, Csf-1R, or Vegfr-2. The functions of those and other receptors in adult RGCs are poorly understood, but these results suggest that RGCs have the capacity to utilize and may be dependent on several RTKs for their normal development and maintenance.
